# *Spp1* Appears to Be a Key Gene for Sporadic Obstructive Hydrocephalus in the Absence of AQP4

**DOI:** 10.3390/ijms262110290

**Published:** 2025-10-22

**Authors:** Miriam Echevarría, Laura Hiraldo-González, José Luis Trillo-Contreras, Francisco D. Rodríguez-Gómez, Francisco Mayo, Elaheh Sobh-Doush, Carmen Ortiz-Salguero, Javier Villadiego, Reposo Ramírez-Lorca

**Affiliations:** 1 Instituto de Biomedicina de Sevilla, IBiS, Hospital Universitario Virgen del Rocío, CSIC, Universidad de Sevilla, 41013 Sevilla, Spain; 2 Departamento de Fisiología Médica y Biofísica, Facultad de Medicina, Universidad de Sevilla, 41009 Sevilla, Spain; 3 Centro de Investigación Biomédica en Red Sobre Enfermedades Neurodegenerativas (CIBERNED), 28029 Madrid, Spain

**Keywords:** congenital hydrocephalus, AQP4 knockout mouse, differential gene expression study, microglia/macrophages, Aquaporin, *Spp1*, osteopontin

## Abstract

Aquaporin-4 (AQP4) is expressed in ependymal cells bordering the ventricles, the glia limitans, and pericapillary astrocyte endfeet forming the blood–brain barrier. The sporadic occurrence of obstructive congenital hydrocephalus (OH) has been observed in the offspring of AQP4^−^/^−^ mice generated in the CD1 strain background. Here, we used microarray analysis to explore gene expression profiles in the periaqueductal area from littermate AQP4^−^/^−^ pups at postnatal day 12. We compared wild-type (WT) animals with AQP4^−^/^−^ animals that developed OH (AQP4^−^/^−^-OH) and those that did not (AQP4^−^/^−^-NH). Bioinformatic analysis identified gene sets associated with proliferation and migration of microglia, ependymal cell adhesion, extracellular matrix components, axon myelination, and neuronal synapsis. Among the differentially expressed genes, *Spp1*—expressed by neonatal CD11c^+^ microglia—was highlighted in the triple comparison. *Spp1* was significantly upregulated in AQP4^−^/^−^-NH and downregulated in AQP4^−^/^−^-OH mice. These findings suggest that CD11c^+^ microglia, via *Spp1* expression, play a key morphogenic role in the aqueduct of Sylvius and their absence, occurring in a small subset of AQP4^−^/^−^-CD1 animals, leads to obstructive hydrocephalus.

## 1. Introduction

In humans, cerebrospinal fluid (CSF) is contained within cavities of the central nervous system (CNS) that are lined with ependymal cell layer [1,2]. These cavities include the cerebral ventricles (the lateral ventricles, the third ventricle, the aqueduct of Sylvius, and the fourth ventricle), the spinal canal, the cistern magna, the cistern pontis, and the subarachnoid space. The total volume of CSF is 150 mL, with a production rate of 550 mL per day. This implies that complete turnover occurs approximately four times per day [3]. Hydrocephalus is a disorder characterized by the excessive accumulation of CSF mainly in the cerebral ventricles. This accumulation normally leads to an increase in intracranial pressure (ICP), which is harmful to brain tissue. Hydrocephalus is significantly associated with high neurological morbidity and mortality; and it can be classified in different ways according to various criteria [4,5]. One distinction is between congenital and acquired hydrocephalus, depending on when it develops. Another distinction is between communicating and non-communicating or obstructive hydrocephalus, depending on whether there is an interruption in the ventricular flow of CSF [5,6]. In communicating hydrocephalus, CSF flow is maintained through the ventricles; the blockage occurs after CSF leaves the ventricles through the Luschka and Magendie foramen. In non-communicating or obstructive hydrocephalus, CSF flow is obstructed at the ventricles. The most common cause is stenosis of the aqueduct of Sylvius [7]. This leads to CSF accumulating in the anterograde regions (the lateral and third ventricles), resulting in ventriculomegaly and CSF transudation.

Aquaporin 4 (AQP4), the most important aquaporin in the brain, is expressed in glial membranes with the highest density over the endfeet membranes of subpial and perivascular astrocytes. AQP4 is also abundantly expressed in ependymal cells that line the ventricular system [8,9]. Numerous studies in recent years have demonstrated the important role of AQP4 in the production and circulation of CSF [10,11,12,13]. In addition to facilitating the efficient movement of fluid between various brain compartments (blood, interstitium, intracellular space, and CSF) as a water channel, AQP4 is an integral membrane protein, in astrocytes and ependyma, that provides structural support to their cellular membranes [8]. The polarization of AQP4 to astrocytic endfeet is mediated by its interaction with α-syntrophin, an intracellular structural protein in the dystrophin-associated protein complex (DAPC), which connects the astrocyte podocyte’s actin skeleton with AQP4 and the extracellular proteins of the basement membrane: laminin and agrin [14,15,16]. Abnormal AQP4 expression or distribution has been associated with hydrocephalus. Experimental models, including hop gait mice with congenital hydrocephalus and rats with hydrocephalus induced by kaolin injection into the cisterna magna, have demonstrated a close association between hydrocephalus and AQP4 expression. In particular, intracerebral kaolin injection induces inflammation and ependymal disruption—conditions under which AQP4 expression is markedly upregulated—suggesting a compensatory role for AQP4 in maintaining water homeostasis and limiting ventricular dilation [17,18,19,20]. Changes in AQP4 expression and in cell-to-cell binding proteins, such as N-cadherin and Connexin 43, at the CNS/CSF interface have been associated with hydrocephalus in humans and animal models [21,22,23]. Consistent with this association, 9% of AQP4^−/−^ (knockout, KO) mice offspring have stenosis of aqueduct of Silvio, which causes death during the first month of life due to obstructive congenital hydrocephalus [24]. Genetic alterations that produce abnormalities in the ependyma have been demonstrated in AQP4-KO-CD1 (AQP4^−/−^) mice [25]. Therefore, to elucidate the differences in the transcriptome between AQP4-KO-CD1 animals that develop obstructive hydrocephalus with that of AQP4-KO-CD1 siblings from the same litter that did not develop hydrocephalus, we performed a study of differential gene expression in the aqueduct of Sylvius of both type of animals, as well as control or wild-type (WT) mice, using microarray technology from Affymetrix. Our analysis was done in mice at 12 days after birth, given that the sporadic obstructive hydrocephalus phenotype was observed in AQP4^−/−^-CD1 mice by Feng et al. [24] at this stage of development. In recent works of our laboratory we had described for the first time that the expression of a microglial subtype, CD11c^+^, plays an important role in the normal formation of aqueduct ependyma in mice [26]. We demonstrated that these microglia cell type highly expressed in the subependymal zone of the aqueduct, remain for a longer time in the periaqueduct area of an AQP4-KO/C57BL/6 animal compare to a WT animal, indicating the crucial role of CD11c^+^ for the development of this periaqueductal tissue [26,27]. The findings that we present here are consistent with those results and actually were obtained before our last works [26,27], but remain unpublished because by the time we finish recollecting the tissue samples we used for the present Affymetrix analysis, we had started outcrossing the AQP4^−/−^-CD1 mice with WT-C57BL/6 animals to obtain a C57BL/6 genetic background of the AQP4-KO animal, and unfortunately by doing that we lost the sporadic obstructive hydrocephalus phenotype, making impossible to validate our results in this type of hydrocephalus animal. Nevertheless, we reconsider this limitation and still believe that the results we present here are extremely relevant for the scientific community, especially for researchers interested in understanding the molecular bases underneath the origin of certain types of congenital hydrocephalus. Recent publications have highlighted the important role of postnatal amoeboid CD11c^+^ microglia in white matter regions such as the corpus callosum, cerebellum, and cortex [28,29,30,31]. These cells display a distinctive transcriptomic profile in which *Spp1* expression appears to play a crucial role in promoting tissue repair and maintaining the structural integrity of the developing brain via Osteopontin (OPN, the protein encoded by the *Spp1* gene) release [32]. In light of these observations, it is conceivable that changes in *Spp1* expression associated with *AQP4* deficiency could affect the normal development of the aqueduct and contribute to the onset of hydrocephalus. A better understanding of the molecular interactions among *AQP4*, *Spp1*, and microglial activity may therefore provide new insights into the pathophysiological mechanisms of congenital hydrocephalus and suggest potential avenues for therapeutic intervention.

## 2. Results

### 2.1. Development of Obstructive Hydrocephalus in AQP4^−^/^−^-CD1 Mice

**Figure 1:**
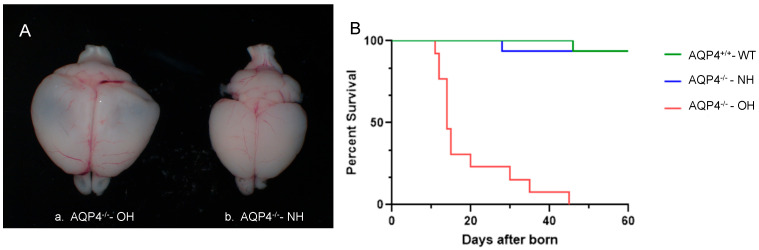
(**A**) Brains dissected from AQP4^−/−^ CD1 mice that either develop obstructive hydrocephalus (AQP4^−/−^-OH) (**a**) or that did not develop hydrocephalus (AQP4^−/−^-NH) (**b**). (**B**) Survival of animals AQP4^−/−^-OH, AQP4^−/−^-NH and wild type (WT), in days, from the day of birth.

While maintaining the AQP4^−/−^/CD1 colony, 9% of the offspring of the AQP4^−/−^ mice sporadically developed hydrocephalus. Animals with hydrocephalus could be distinguished by eye due to clear characteristics such as reduced body size, an enlarged head and gait disturbances. These differences were observed as early as postnatal day 7 (P7). Dissected brains showed a large accumulation of liquid, measuring more than twice the volume, in AQP4^−/−^-OH animals compared to AQP4^−/−^-NH animals (Figure 1A). Furthermore, the survival rate of hydrocephalic animals was significantly lower (Figure 1B). This was the reason why the animals were sacrificed between days 11 and 12 after birth, as previously described by other authors [24]. The survival record (Figure 1B) confirmed previous descriptions [24]; that WT and AQP4^−/−^-NH mice did not differ in survival time during the first 60 days of life. Meanwhile, the survival rate of AQP4^−/−^-OH mice that developed symptoms of hydrocephalus early in the first postnatal week dropped drastically to 50% by days 11–13, with all animals dying before reaching 50 days of age.

### 2.2. Microarray-Based Differential Gene Expression Study

**Figure 2:**
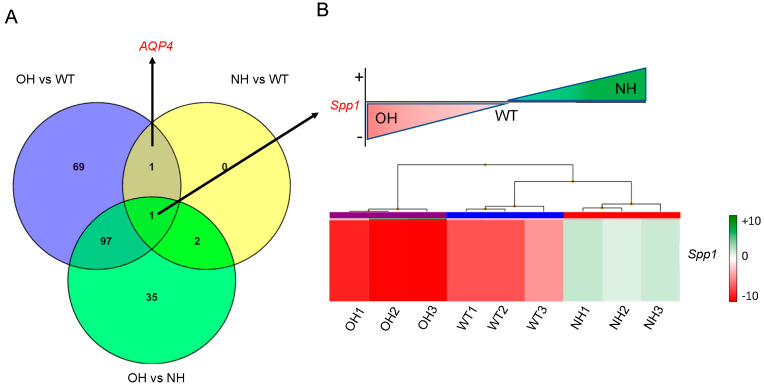
Triple comparison between AQP4^−/−^-obstructive hydrocephalus mice (OH), AQP4^−/−^-not hydrocephalus mice (NH) and wild-type animals (WT). (**A**) Venn diagram. This diagram shows the overlap between groups of differentially expressed genes (FC > 2 or <−2; and FDR < 0.05) in the comparison performed. Each circle represents a comparison. The number of differentially expressed genes appears inside each area. This figure was created using Venny 2.118, a free online web tool. (**B**) Heat map for *Spp1* in the three groups of compared animals was created by Transcriptome Analysis Console (TAC v4.0.3.14) software.

The Affymetrix study results revealed significant heterogeneity in the gene profiles of the individual samples. This divergence, combined with the small sample size, was likely responsible for the low number of differentially expressed genes that exceeded the imposed statistical thresholds. The sample size (N = 3) refers to biological replicates for each group used in the microarray experiment (WT, AQP4^−^/^−^-OH, and AQP4^−^/^−^-NH). This number was selected according to standard requirements for microarray-based gene expression profiling, where each replicate represents pooled tissue from three littermate pups. Genes designated as differentially expressed had an FDR (false discovery rate, a correction of the conventional *p*-value that reduces the probability of false positives by 1 × 10^6^) less than 0.05 and an FC greater than 2.0 (for activated genes) or less than −2.0 (for repressed genes) in at least one of the three comparisons. Once all the results obtained from the microarray study were analyzed, it was interesting to determine whether there was overlap between the groups of genes that are differentially expressed in each case. To do this, a Venn diagram was created using the free online tool Venny 2.118 [33]. As shown in Figure 2A, a total of 205 genes were differentially expressed when considering all possible comparisons. In the comparison between AQP4^−/−^-NH and WT animals, one gene, Aqp4, showed a significant degree of inhibition, while three genes, *Spp1*, *Ccl9* and *Atp6v0d2*, were identified as being upregulated. When the comparison was done between AQP4^−/−^-OH and WT, 168 genes were differentially expressed, and 135 genes resulted differentially expressed when the two knockout animals, AQP4^−/−^-NH and AQP4^−/−^-OH, were contrasted. After overlapping these comparisons by pairs, *AQP4* was identified as the only gene consistently differentially expressed between WT and both knockout groups, and only one gene, *Spp1*, was observed in the central zone for overlapping of the three double comparisons (Figure 2A). Upon closer examination of *Spp1* expression levels per group (N = 3, with three animals per group), in each condition (AQP4^−/−^-OH, AQP4^−/−^-NH, and WT), it was found that *Spp1* expression was higher in AQP4^−/−^-NH animals than in WT animals, and higher in WT animals than in AQP4^−/−^-OH mice. In other words, AQP4^−/−^-OH animals showed significant downregulation of *Spp1* levels compared to WT animals, and this downregulation was further accentuated when compared to AQP4^−/−^-NH animals (Figure 2B). Table 1 shows five of the more differentially expressed genes, which have an FC value greater than 2 and an FDR value less than 0.05, in two of the comparisons performed. These genes behave similarly to *Spp1*. That is, they were upregulated in AQP4^−/−^-NH animals compared to WT and AQP4^−/−^-OH animals and downregulated in AQP4^−/−^-OH animals compared to WT and AQP4^−/−^-NH animals. As seen in Table 1, all the genes (*Spp1, Ccl9, Atp6v0d2, Cpxm2, Gpnmb,* and *Itgax*) were upregulated in the AQP4^−/−^-HC vs. WT comparison and downregulated in the AQP4^−/−^-OH vs. WT comparison.

### 2.3. Gene Set Enrichment Analysis

**Figure 3:**
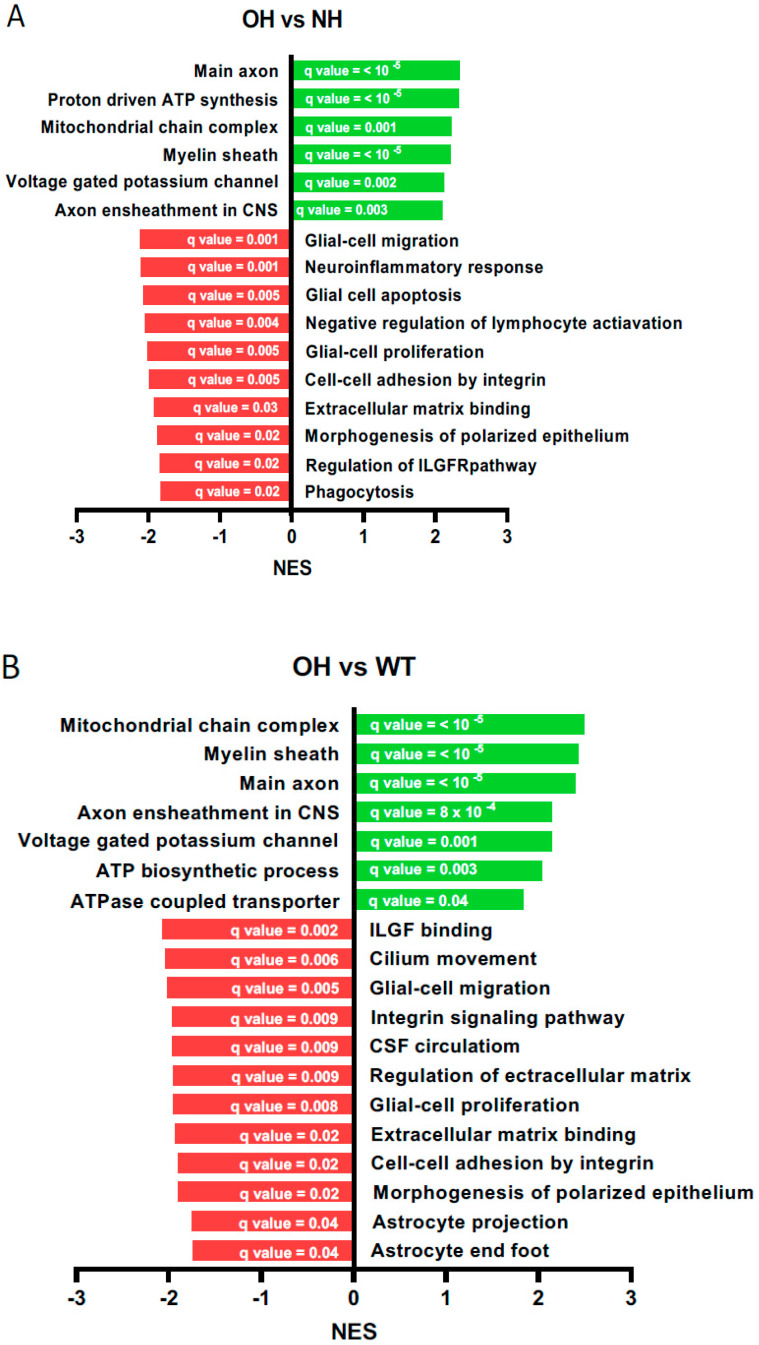

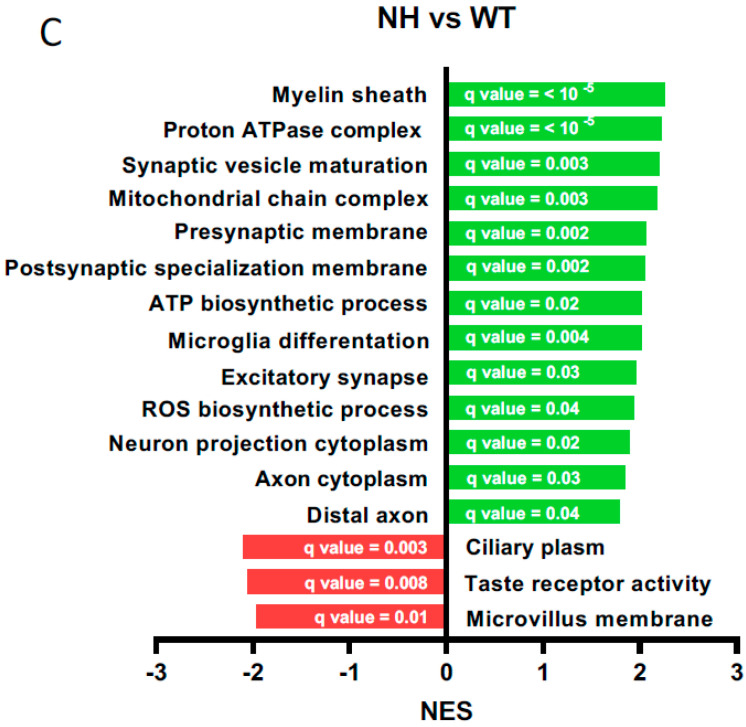
Gene Ontology terms upregulated or downregulated in the different comparisons performed: (**A**) Obstructive hydrocephalus animals, AQP4^−/−^-OH, (OH) versus No hydrocephalus mice AQP4^−/−^-NH, (NH), (**B**) Obstructive hydrocephalus animals AQP4^−/−^-OH, (OH) versus wild-type mice (WT), and (**C**) No hydrocephalus mice, AQP4^−/−^-OH (OH) versus WT. Gene Set Enrichment Analysis (GSEA) was performed using a pre-ranked list of genes sorted by *p* value from most significantly upregulated to most significantly downregulated in the different comparisons shown. Normalized Enrichment Score (NES) is represented in the *X* axis, and the q value is indicated inside each bar.

A gene set enrichment analysis (GSEA) using the gene ontology database was performed after bioinformatically processing and filtering the raw data obtained in the microarray analysis [34,35]. This process yielded changes in gene sets (GS), which, when integrated under the same pathway and with alterations in the same direction (i.e., an increase or decrease in expression), suggested alterations in specific biological processes, molecular functions, or cellular components among the evaluated conditions in terms of enrichment (normalized enrichment score, NES). Figure 3 shows the main ontological terms that were statistically significant in the three comparisons performed. In the comparison between obstructive hydrocephalus and non-hydrocephalus KO animals (AQP4^−/−^-OH vs. AQP4^−/−^-NH), the most increased ontological pathways were those associated with structural elements of axons and myelin sheaths, mitochondrial respiratory chain complexes, and ATP synthesis, as well as voltage-gated potassium channels (Figure 3A). Conversely, a long list of decreased pathways was observed in this comparison (see Supplementary Table S1), which can be grouped into two or three main categories. One category is associated with cells, processes, and mechanisms involved in the immune system; other category includes morphogenesis of the epithelium, cell-to-cell adhesion and extracellular matrix binding, and the other category includes components of the insulin-like growth factor receptor (ILGFR) pathways (Figure 3A). For the comparison of obstructive hydrocephalus versus wild type (AQP4^−/−^-OH vs. WT), the analysis of affected functional enrichment and ontological terms revealed a similar situation to the previous comparison (Figure 3B). This analysis again showed that proteins associated with the mitochondrial chain and ATP synthesis were increased in the hydrocephalic animal, as well as elements related to axon myelination and neuronal potassium transporter conductance. In this comparison, again a long list of pathways, processes and cell types related to the immune system (lymphocytes and glia migration and proliferation, integrin signaling), Ilgf signaling, extracellular matrix components and cell–cell epithelium conformation as well as normal astrocytes end foot projection were found down regulated in the hydrocephalus animal AQP4^−/−^-OH compared to the WT (Figure 3B and Supplementary Table S2). Finally, the analysis where non-hydrocephalus-AQP4^−/−^-NH animals were compared to the WT ones (Figure 3C), showed a long list of ontological pathways increased in the AQP4^−/−^-NH animals, that curiously contrasted with the short list of reduced elements or functions found in the AQP4^−/−^-NH animals respect to the WT ones. Among the upregulated paths, besides those that were previously described for the hydrocephalus-AQP4^−/−^-OH animal, such as myelin sheath and elements related with myelination, axon components, mitochondrial chain complex and ATP production, two novel pathways stand out as highlights among the overexpressed processes. One is associated with an increase in the biosynthesis of superoxide free radicals (ROS), and the other implies higher levels of microglia differentiation (Figure 3C and Supplementary Table S3). By contrast, cilium, flagellum, and microvillus elements of the ependyma were found reduced in the AQP4^−/−^-NH animals as well as circulation of the CSF; and sensorial functions as the taste receptor activity were found reduced in the AQP4^−/−^-NH animals compared to WT ones (Figure 3C and Supplementary Table S3).

## 3. Discussion

Over 15 years have passed since Feng et al. [24], reported the unexpected occurrence of sporadic cases of obstructive hydrocephalus in a small percentage of newborn AQP4^−/−^ animals. Examination of a subset of encephalomegaly mice revealed severe ventricular hydrocephalus and elevated intracranial pressure. These mice died by six weeks of age, with a median survival time of 28 days. Initial explanations for the etiology of the described hydrocephalus included ependymal disorganization causing aqueduct adhesion. However, the precise link between the lack of AQP4 and ependymal cell abnormalities remained unclear. Another possibility suggested is the existence of a modifier gene that primes the brain for hydrocephalus development in the absence of AQP4 [24]. However, identifying such a gene would be very challenging. The work presented here aimed to understand the underlying molecular basis of the sporadic hydrocephalus described above. To this end, we received the KO animal developed in doctor Verkman’s laboratory, which are mice with the same genetic background as those described by Feng et al. [24]. In our hands the record of the animals’ survival (Figure 1) did confirm what had been previously described [24], which was that WT and AQP4^−/−^-NH mice did not differ in survival time, at least during the first 60 days of life. Meanwhile, the survival rate of AQP4^−/−^-OH mice that developed symptoms of hydrocephalus early, by the first postnatal week, dropped drastically to 50% by days 11–13; all died before reaching 50 days of age. Similarly to the original transgenic colony, the appearance of the hydrocephalus condition was exclusively observed in animals homozygous for the mutation. No sporadic hydrocephalus phenotype was ever observed in heterozygous (AQP4^+/−^) or WT animals (AQP4^+/+^). Important enlargement of the head and ventriculomegaly, as well as difficulty walking, were characteristics clearly distinguishable in AQP4^−/−^-OH animals at a very early age, i.e., by postnatal days 7–11, as illustrated in Figure 1. Interestingly, however, the incidence of hydrocephalus was around 9% at the beginning of colony establishment. Over time, this percentage gradually decreased until the obstructive hydrocephalus phenotype was completely lost. Shortly after the Affymetrix analysis was completed, the genetic background of the AQP4^−/−^ colony was C57/BL6, rather than CD1, as at least ten rounds of backcrossing with C57/BL6 parents had been performed. Thus, the only explanation for the loss of the hydrocephalus phenotype appears to be the different genetic background. Abundant differences in enzymes and metabolic processes have been reported among these two strains of mice [36]. These differences somehow will be responsible for a mild penetrance of the *AQP4* gene deletion in the C57/BL6 mice that does not result in the detrimental effect observed in the CD1 mouse. Further analysis of AQP4 deletion in both strains of mice involving gene expression and cell regulatory processes will be necessary to understand why the obstructive hydrocephalus condition appears in CD1 animals but not in C57/BL6 mice. On the other hand, while the loss of the obstructive hydrocephalus phenotype was interesting from a genetic standpoint, it significantly limited the scope of the initial analysis intended, since validating the Affymetrix results using alternative techniques (RT-qPCR and immunohistochemistry) in additional samples was not possible. Future work will include verification of *Spp1* expression and Cd11c^+^ microglia presence once a new colony expressing the hydrocephalus phenotype is available.

Transcriptomic analysis of the aqueduct samples revealed differentially expressed genes and pathways in the three animals analyzed. The triple comparison shown in the Venn diagram (Figure 2A), surprisingly identified the *Spp1* gene as the only one that appeared to be differentially expressed when comparing, by pairs, the gene expression levels of AQP4^−/−^ mice with obstructive hydrocephalus (OH) or without hydrocephalus (NH), and wild-type animals (WT). Assuming the levels in the WT animal to be the control ones, Affymetrix expression analysis revealed that this gene was significantly downregulated in the animals that developed obstructive hydrocephalus AQP4^−/−^-OH (Figure 2B). Conversely, it was significantly upregulated in the animals that did not develop hydrocephalus AQP4^−/−^-NH and exhibited an apparently normal phenotype (Figure 2B).

A notable result was that the overlapping regions between the AQP4^−/−^-OH vs. WT and AQP4^−/−^-NH vs. WT comparisons were very small. In fact, they only share *Aqp4*, since both animals (OH and NH) were AQP4 knockout, and the *Spp1* gene as differentially expressed (Figure 2A). Similarly, the large number of differentially expressed genes found in the AQP4^−/−^-OH vs. AQP4^−/−^-NH comparison was noteworthy, given that both animals have the same genetic background. The functional annotation of repressed genes in the AQP4^−/−^-OH vs. AQP4^−/−^-NH comparison stands out, raising the hypothesis that a mechanism, probably linked to the immune system (Figure 3A), is responsible for the large phenotypic differences between AQP4^−/−^-NH and AQP4^−/−^-OH animals. In this scenario, *Spp1* would be positioned as the key gene orchestrating the amelioration of congenital hydrocephalus because it is over-expressed only in animals AQP4^−/−^-NH that did not develop hydrocephalus. Conversely, lack of *Spp1* gene during the first postnatal days (P0-P11) would be a critical condition that primes the brain for the development of hydrocephalus in the absence of AQP4.

**Figure 4:**
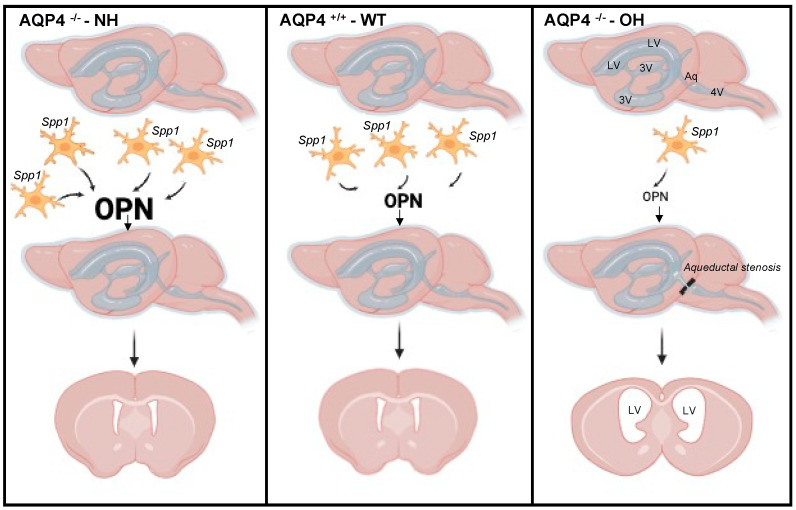
This is a summary of the events that occur in the three different animal models: AQP4^−^/^−^ mice with obstructive hydrocephalus (OH), AQP4^−^/^−^ mice without hydrocephalus (NH), and wild-type (WT) animals. In the NH animals, a significant number of reactive microglia expressing *Spp1*, arrive in the aqueductal area and release a significant amount of osteopontin. This compensates for the lack of AQP4, enabling the aqueduct of Sylvius to develop normally, as in WT animals. This prevents obstructive hydrocephalus, which is observed in OH animals. In OH animals, *Spp1* expression and the amount of OPN released are both reduced. A presumed stenosis of the aqueduct of Sylvius in these animals would cause aqueductal obstruction and enlargement of the lateral and third ventricles due to CSF accumulation (obstructive hydrocephalus). LV: lateral ventricle; 3V: third ventricle, 4V: fourth ventricle, Aq: Aqueduct of Sylvius, *Spp1*: secreted phosphoprotein 1, OPN: Osteopontin (coding protein of *Spp1*), 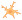 reactive microglia.

Identifying the cellular source of the *Spp1* gene in the AQP4^−/−^-NH/CD1 mouse was impossible, given the loss of the obstructive hydrocephalus phenotype. Nevertheless, microglia cells are the most likely cell type responsible for *Spp1* expression. This fits with the significant immune system impairment observed in the hydrocephalic animal, as deduced from the extensive list of downregulated processes and properties associated with lymphoid and glial cells, including cell migration, proliferation, phagocytosis, apoptosis, inflammation, chemokine binding, and ILGF signaling (Figure 3A and Supplementary Table S1). Recent publications have demonstrated the important role of postnatal amoeboid microglia in white matter, including the corpus callosum, cerebellum, and cortex [28,29,30,31], which display a very particular transcriptomic state, out of which expression of *Spp1* seems to have a crucial role in contributing to the rapid repair of cavitary lesions and maintaining the structural integrity of the developing brain during normal morphogenesis [32]. Our work, in AQP4-KO-C57/BL6 animals [26,27], has demonstrated the key role of a postnatal population of CD11c^+^ microglia with durable *Spp1* expression, in the normal morphogenesis of the aqueduct of Sylvius. We hypothesized therefore, that similar cellular process will occur in most (>90%) of AQP4^−/−^/CD1 mice, and the presence of a CD11c^+^ microglia with overexpression of *Spp1* near the aqueduct of Sylvius might contribute to a protective mechanism reducing the likelihood of aqueductal obstruction in AQP4^−^/^−^ mice. The failure of the cD11c^+^ subtype of microglia to arrive at the aqueduct zone during the first postnatal week, for reasons that are still unknown, seems to occur in less than 9% of newborn homozygotes for the AQP4 gene mutation. This failure might result in the development of obstructive hydrocephalus in these mice; however, this interpretation should be considered preliminary and warrants further validation. Figure 4 illustrates the three animal models described here: WT, AQP4^−/−^-NH and AQP4^−/−^-OH. The Affymetrix results also showed overexpression of genes such *Ccl9*, *Gpnmb*, *Itgax* and *Cpxm2*, that likewise *Spp1*, form part of the transcriptomic signature of certain postnatal subtypes of microglia distinctly named as axon tract-associated microglia (ATM) [37], proliferative-region associated microglia (PAM) [30], youth-associated microglia (YAM) [32], or CD11c^+^ microglia [29,37,38]. For those genes (Table 1), the expression pattern was like that described for *Spp1*; all of them were overexpressed in the AQP4^−/−^-NH/CD1 mice without hydrocephalus (AQP4^−/−^-NH), whereas they were downregulated in animals that developed obstructive hydrocephalus (AQP4^−/−^-OH). These findings provide additional support for the significant role in the final formation of the aqueduct of Sylvius of a postnatal CD11c^+^ microglia population that releases multiple anti-inflammatories, neuroprotective and reparative cytokines that prevent malformations and other neural disorders.

In line with this, processes necessary for the normal formation of the ependymal membrane in the aqueduct such as, morphogenesis of polarized epithelium, binding to the extracellular matrix and cell–cell adhesion by integrins, appear among the ontological pathways most downregulated in the hydrocephalus animal (Figure 3A,B). Microvillus and ciliary plasma membrane are components downregulated here in the no hydrocephalus AQP4^−/−^-NH mouse compared to the WT animal (Figure 3C and Supplementary Table S3), sustaining again that affectation of the aqueduct ependyma membrane and its cilia might result in stenosis of the aqueduct of Sylvius and development of obstructive hydrocephalus in some cases. The Axdnd1 gene, which encodes the axonemal dynein light chain domain 1—crucial for ciliary propulsion of cerebrospinal fluid (CSF) [39,40], was downregulated in both AQP4^−^/^−^-OH and AQP4^−^/^−^-NH mice (Tables S2 and S3). This reduction may hinder ciliary motility and contribute to CSF stagnation in the ventricular system.

Similar results were shown in AQP4-KO-C57/BL6 mice, in which levels of numerous genes such as *Hydin*, *Cfap43*, *Cfap69*, *Ccdc170* and *Cdhr4*, associated with ciliary proteins and involved in intercellular junction complexes of the aqueduct ependymal membrane, were also downregulated [26,27]. Morphological and functional alterations of the aqueduct ependyma revealed a disorganized ependyma in these AQP4^−/−^ mice, with changes in the intercellular complex union, unevenly orientated cilia, and variations in the planar cell polarity of the apical membrane, that translate into reduced cilia beat frequency, which might alter cerebrospinal fluid movement and eventually lead to development of hydrocephalus.

In contrast to the gene expression downregulation observed in ependyma membrane components and processes, a few ontological pathways appear overexpressed in the AQP4^−/−^/CD1 animals respect to WT ones. Those are believed to represent compensatory responses to diminish the impact for AQP4 deletion over crucial process as neurogenesis and correct neuronal maturation as well as olygodendrogenesis and myelinization process [27,41,42,43,44,45]. Among them, of note is the extensive involvement of structural components of neurons related to pre- and postsynaptic membranes and specific elements of excitatory synapses and axon components (Figure 3A–C, and Supplementary Tables S1–S3). Voltage gate potassium channel activity was also increased, probably to favor the syphoning of excess of this extracellular ion, released by highly activated excitatory synapsis, back to astrocytes deficient in the AQP4 protein [46]. Additionally, significant overexpression of myelin sheath related elements is observed, as well as pathways associated with ATP synthesis and an increase in mitochondrial and proton ATPase complexes. These are most likely associated with the increased cerebral metabolic expenditure resulting from excessive myelin production in the AQP4^−/−^ animal, which would be probably caused by a dysregulated myelination process [45,47]. In line with this, Table 1 shows that the *Atp6V0d2* gene is among the most highly expressed genes in the AQP4^−/−^ animal (NH), although it is strongly downregulated in those mice that develop obstructive hydrocephalus (OH). The *Atp6V0D2* gene codes for a vacuolar-type proton ATPase that contributes to proton transport to the lysosome. It is highly expressed by macrophages and contributes to myelin degradation and removal in nerve regeneration during neural repair after injury [48]. Previous studies have demonstrated alterations into the myelinization process in the AQP4^−/−^/C57BL/6 animal [27,47]. In these animals, increased expression of oligodendrocyte precursor marker genes was detected; however, this was not accompanied by a rise in mature oligodendrocyte abundance, and axonal myelination in the corpus callosum was reduced relative to WT mice. Therefore, the findings in AQP4^−^/^−^ CD1 animals may reflect concurrent alterations in myelination and neuronal developmental processes.

Taken together, our findings indicate that AQP4 deficiency alters the molecular and cellular environment around the Sylvian aqueduct, compromising ependymal integrity and microglial reparative activity, which may predispose to aqueductal stenosis and hydrocephalus in a subset of animals. AQP4 is a crucial molecular component of the glymphatic pathway, which facilitates interstitial fluid and solute clearance from the brain parenchyma. Alterations in AQP4 localization or expression have been linked to impaired glymphatic function, resulting in the accumulation of metabolic waste products and contributing to neurodegenerative and age-related brain disorders [49,50]. Therefore, the loss of AQP4 in our model may not only compromise ependymal integrity but also disrupt glymphatic circulation, further altering cerebrospinal fluid dynamics. These combined mechanisms may underlie, at least in part, the susceptibility to aqueductal obstruction and hydrocephalus observed in AQP4^−^/^−^ animals.

## 4. Materials and Methods

### 4.1. Animals

AQP4^−/−^/CD1 mice (kindly provided by Dr. A. Verkman, UCSF, San Francisco, CA, USA) and wildtype (WT) littermates were housed at a controlled temperature (22 ± 1 °C) in a 12 h light/dark cycle, with ad libitum access to food and water. The AQP4^−/−^ mice were genotyped as indicated previously for AQP4 [51]. We established our own colony of AQP4^−/−^ mice by crossing them with WT animals. However, instead of using CD1 animals as in the original strain, we began rederivating the KO genotype to the C57/BL6 strain. It took more than a year to collect the periaqueductal tissue from the three animals of interest, all siblings, obtained from the same colony: WT, AQP4^−/−^-NH and AQP4^−/−^-OH. The mice were sacrificed under anesthesia using a combination of 100 mg/kg of ketamine (Pfizer, Inc., New York, NY, USA) and 10 mg/kg of xylazine (Bayer, AG, Leverkusen, Germany). The animals were sacrificed at P12 for RNA extraction for microarray analysis. All experiments were performed in accordance with European Directive 2010/63/EU and Spanish Royal Decree 53/2013 on the protection of animals used for scientific purposes. The study was approved by the Animal Research Committee of the Virgen del Rocío University Hospital (approval number 26/01/2017/017; approval date: 26 January 2017; University of Seville).

### 4.2. RNA Purification

For the microarray study, periaqueductal (Aqueduct of Sylvius, Aq) samples were taken from the brains of AQP4^−/−^/CD1 mice at P12 (postnatal day 12), with obstructive hydrocephalus phenotype (AQP4^−/−^-OH) or without hydrocephalus (AQP4^−/−^-NH), and from wild-type (WT) mice. Total RNA purification from tissue samples was performed using the commercial Qiagen^®^ RNAeasy micro kit 50 (Cat. No. 74004; Qiagen GmbH, Hilden, Germany). This kit is based on cell lysis with guanidine isothiocyanate and subsequent RNA purification using silica adsorption columns. After obtaining the final purified RNA, the concentration was measured, and contamination by organic solvents (A260/A230) and proteins (A260/A280) was assessed by spectrophotometric analysis on a NanoDrop™ 2000/2000c Spectrophotometer (Thermo Fisher Scientific, Waltham, MA, USA).

### 4.3. Differential Gene Expression by Microarray

To screen for differential gene expression under different conditions, a microarray study was conducted using Affymetrix^®^ Mouse Clariom™ S Assay GeneChip (Thermo Fisher Scientific, Waltham, MA, USA). This technology is based on the fact that the expression levels of a gene are directly proportional to the detectable transcripts (mRNA) of that gene in a sample. Microarrays are devices that have probes arranged in a matrix, DNA sequences complementary to the sequences of the different known transcripts in a species. Once the total RNA has been extracted from a sample, it is labelled, typically with fluorescence, and incubated on the microarray support so that it hybridizes with the specific probes. After a series of washes, expression levels are estimated by measuring the fluorescence of the transcripts that have hybridized with the probes. The microarray study was carried out by the Genomics and Sequencing Service of the Institute of Biomedicine of Seville using aqueduct samples supplied in RNAlater. Three independent samples per condition were analyzed, and periaqueductal tissue from N = 3 animals were pooled on each of the three samples, summarizing a total of N = 9 animals analyzed per experimental condition compared. The comparisons made were between the three different conditions: AQP4^−/−^ CD1 mice with obstructive hydrocephalus (AQP4^−/−^-OH) versus WT mice; WT mice versus CD1 mice without hydrocephalus (AQP4^−/−^-NH) and AQP4^−/−^ CD1 mice with obstructive hydrocephalus (AQP4^−/−^-OH) versus AQP4^−/−^ CD1 mice without hydrocephalus (AQP4^−/−^-NH).

### 4.4. Statistical Analysis

For the analysis of gene levels and differential expression, Venn diagrams, and heatmaps, the Affymetrix^®^ Transcriptome Analysis Console (TAC v4.0.3.14) software was used to perform statistical tests. To perform a gene set enrichment analysis (GESEA) we used a preranked list of genes according to the −log10 of the *p*-value multiplied by the sign of the fold-change. This preranked list was analyzed using the GENE SET ENRICHMENT ANALYSIS 4.3.1 software [52], with a maximum size of 1000 and a minimum size of 5, against the Gene Ontology database [53,54], retrieved from the Molecular Signatures Database (MSigDB) repository [55] on 27 June 2025.

## 5. Conclusions

In summary, our findings suggest that the development of obstructive hydrocephalus in a small proportion of AQP4^−^/^−^/CD1 mice may be associated with a reduced presence of CD11c^+^ microglia and lower *Spp1* (osteopontin) expression near the aqueduct of Sylvius during early postnatal development. In contrast, most AQP4^−^/^−^ animals exhibit an abundant arrival of CD11c^+^ microglia to the aqueductal region, leading to increased *Spp1* expression and the release of other reparative cytokines that may help preserve ependymal integrity and prevent aqueductal obstruction. These observations point toward a potential morphogenic and protective role of microglial *Spp1* in maintaining aqueductal patency. However, given the limited sample size and the correlative nature of these findings, further studies are required to confirm this mechanism and to clarify how AQP4 deficiency interacts with microglial function and ependymal maintenance.

## Figures and Tables

**Table 1:** Genes differentially expressed (with FC > 2 or <−2, and *p*-value < 0.05), for the comparisons AQP4^−/−^-OH vs. AQP4^−/−^-NH and AQP4^−/−^-NH vs. WT. Note that all genes indicated have the same tendency as that observed for *Spp1*, which is that genes are downregulated in the AQP4^−/−^-OH animal compared to the AQP4^−/−^-NH ones, but they were upregulated in AQP4^−/−^-NH animals compared to WT ones.

Gene Symbol	Gene Name		OH vs. NH	OH vs. WT	NH vs. WT
***Spp1***	Secreted phosphoprotein 1	FC	−149.57	−3.52	42.47
		*p*-value	8.87 × 10^−9^	3.0 × 10^−4^	1.51 × 10^−7^
***Ccl9***	Chemokine (C-C motif) ligand 9	FC	−6.06	−1.72	3.52
		*p*-value	2.27 × 10^−8^	1.3 × 10^−3^	2.20 × 10^−7^
***Atp6V0d2***	ATPase H+ Transporting V0 Subunit D2	FC	−10.52	−1.94	5.43
		*p*-value	1.86 × 10^−6^	0.016	1.72 × 10^−5^
***Cpxm2***	Carboxypeptidase X, M14 Family Member 2	FC	−4.09	−1.38	2.97
		*p*-value	1.74 × 10^−5^	ns	3.01 × 10^−5^
***Gpnmb***	Glycoprotein (transmembrane) nmb	FC	−17.45	−1.93	9.03
		*p*-value	2.09 × 10^−5^	0.039	2.0 × 10^−4^
***Itgax***	Integrin alpha X	FC	−2.44	1.11	2.7
		*p*-value	3.0 × 10^−4^	ns	3.60 × 10^−5^

## Data Availability

The raw data supporting the conclusions of this article will be made available by the authors on request.

## Supplementary Materials

The following supporting information can be downloaded at: https://www.mdpi.com/article/10.3390/ijms262110290/s1.
