# Supplementary material for: Spp1 Appears to Be a Key Gene for Sporadic Obstructive Hydrocephalus in the Absence of AQP4
Source: Int J Mol Sci. 2025 Oct 22;26(21):10290. doi: 10.3390/ijms262110290 (PMC12609145; doi:10.3390/ijms262110290)
Supplement: Supplementary file 1 [file ijms-26-10290-s001.zip › ijms-3917018-supplementary.pdf]

Article

Spp1 appears to be a key gene for sporadic obstructive hydrocephalus in the absence of AQP4

Miriam Echevarría [1,2,*], Laura Hiraldo-González [1,2], José Luis Trillo-Contreras [1,2], Francisco D. Rodríguez-Gómez [2], Francisco Mayo [1,2], Elaheh Sobh-Doush [1,2], Carmen Ortiz-Salguero [1,2], Javier Villadiego [1,2,3] and Reposo Ramírez-Lorca [1,2,*]

[1] Instituto de Biomedicina de Sevilla, IBiS, Hospital Universitario Virgen del Rocío, CSIC, Universidad de Sevilla, 41013 Sevilla, Spain; laurahiraldo.g@gmail.com (L.H.-G.); josetricon@gmail.com (J.L.T.-C.); francisco.mayo-leon@ec.europa.eu (F.M.); elasob@alum.us.es (E.S.-D.); carmen.corita@gmail.com (C.O.-S.); fvilladiego@us.es (J.V.)

[2] Departamento de Fisiología Médica y Biofísica, Facultad de Medicina, Universidad de Sevilla, 41009 Sevilla, Spain; frandirogo@hotmail.com

[3] Centro de Investigación Biomédica en Red Sobre Enfermedades Neurodegenerativas (CIBERNED), Madrid, Spain

* Correspondence: irusta@us.es (M.E.); reporamirez@us.es (R.R.-L.); Tel.: +34-955923036

Table S1. *Gene Set Enrichment Analysis (GSEA) of periaqueductal tissue from $AQP4^{-/-}$ mice with obstructive hydrocephalus ($AQP4^{-/-}$-OH) compared to $AQP4^{-/-}$ mice without hydrocephalus ($AQP4^{-/-}$-NH).*

OH vs NH				
	**Description**		**FDR q-val**	**NES**
	MAIN AXON	GO:0044304	< 1.0E-5	2,34
	PROTON MOTIVE FORCE DRIVEN ATP SYNTHESIS	GO:0015986	< 1.0E-5	2,32
	MITOCHON. RESPIRATORY CHAIN COMPLEX ASSEMBLY	GO:0033108	0,0014	2,22
	MYELIN SHEATH	GO:0043209	< 1.0E-5	2,211
	NADH DEHYDROGENASE COMPLEX ASSEMBLY	GO:0010257	0,0034	2,12
	VOLTAGE GATED POTASSIUM CHANNEL ACTIVITY	GO:0005249	0,0019	2,12
	AXON INITIAL SEGMENT	GO:0043194	3.59E-4	2,11
	AEROBIC ELECTRON TRANSPORT CHAIN	GO:0019646	0,0042	2,10
	OXIDATIVE PHOSPHORYLATION	GO:0006119	0,0037	2,09
	AXON ENSHEATHMENT IN CENTRAL NERVOUS SYSTEM	GO:0032291	0,0031	2,09
Activated	PROTEIN LOCALIZATION TO AXON	GO:0099612	0,0471	1,94
	GLIAL CELL MIGRATION	GO:0008347	0,0012	-2,11
	NEUROINFLAMMATORY RESPONSE	GO:0150076	0,0018	-2,10
	GLIAL CELL APOPTOTIC PROCESS	GO:0034349	0,0056	-2,06
	INTEGRIN BINDING	GO:0005178	0,0032	-2,06
	NEGATIVE REGULATION OF LYMPHOCYTE ACTIVATION	GO:0051250	0,0042	-2,04
	NEGATIVE REGULATION OF B CELL ACTIVATION	GO:0050869	0,0036	-2,03
	ACTIVATED T CELL PROLIFERATION	GO:0050798	0,0039	-2,01
	GLIAL CELL PROLIFERATION	GO:0014009	0,0037	-2,01
	CELL–CELL ADHESION MEDIATED BY INTEGRIN	GO:0033627	0,0054	-1,99
	NEGATIVE REGULATION OF IL 10 PRODUCTION	GO:0032693	0,0076	-1,96

Repressed	REGULATION GLIAL CELL APOPTOTIC PROCESS	GO:0034350	0,0116	-1,93
	EXTRACELLULAR MATRIX BINDING	GO:0050840	0,0272	-1,92
	CELL ADHESION MOLECULE BINDING	GO:0050839	0,0225	-1,92
	GLIAL CELL ACTIVATION	GO:0061900	0,0130	-1,92
	REGULATION EXTRACELL MATRIX ORGANIZATION	GO:1903053	0,0128	-1,91
	MORPHOGENESIS OF A POLARIZED EPITHELIUM	GO:0001738	0,0173	-1,87
	INSULIN LIKE GROWTH FACTOR (ILGF) I BINDING	GO:0031994	0,0297	-1,87
	REGULATION ILGF RECEPTOR SIGNALING	GO:0043567	0,0226	-1,83
	PHAGOCYTOSIS	GO:0006909	0,0230	-1,83
	PROTEIN COMPLEX INVOLVED IN CELL ADHESION	GO:0098635	0,0471	-1,81
	INTERSTITIAL MATRIX	GO:0005614	0,0420	-1,79

The table lists significantly enriched Gene Ontology (GO) terms identified by GSEA using the preranked gene list from the OH vs NH comparison. Activated (positively enriched) and repressed (negatively enriched) biological processes are shown with their GO identifiers, false discovery rate (FDR q-value), and normalized enrichment score (NES). Only gene sets with FDR q < 0.05 are included.

Table S2. *Gene Set Enrichment Analysis (GSEA) of periaqueductal tissue from $AQP4^{-/-}$ mice with obstructive hydrocephalus ($AQP4^{-/-}$-OH) compared to wild-type (WT) mice.*

OH vs WT				
	Description		FDR q-val	NES
Activated	MITOCHON. RESPIRATORY CHAIN COMPLEX ASSEMBLY	GO:0033108	< 1.0E-5	2,50
	MYELIN SHEATH	GO:0043209	< 1.0E-5	2,43
	MAIN AXON	GO:0044304	< 1.0E-5	2,40
	AEROBIC ELECTRON TRANSPORT CHAIN	GO:0019646	< 1.0E-5	2,25
	NADH DEHYDROGENASE COMPLEX ASSEMBLY	GO:0010257	< 1.0E-5	2,22
	MITOCHONDRIAL GENE EXPRESSION	GO:0140053	4.16E-4	2,18
	AXON ENSHEATHMENT IN CENTRAL NERVOUS SYSTEM	GO:0032291	8.27E-4	2,14
	VOLTAGE GATED POTASSIUM CHANNEL ACTIVITY	GO:0005249	0,0010	2,14
	AXON INITIAL SEGMENT	GO:0043194	0,0000	2,12
	PROTEIN LOCALIZATION TO AXON	GO:0099612	0,0017	2,07
	ATP BIOSYNTHETIC PROCESS	GO:0006754	0,0028	2,04
	CENTRAL NERVOUS SYSTEM MYELIN FORMATION	GO:0032289	0,0236	1,88
	ATPASE COUPLED TRANSPORTER ACTIVITY	GO:0042626	0,0463	1,83
	INSULIN LIKE GROWTH FACTOR I BINDING	GO:0031994	0,0019	-2,07
	CILIUM MOVEMENT	GO:0003341	0,0061	-2,03
	GLIAL CELL MIGRATION	GO:0008347	0,0056	-2,01
	CILIUM OR FLAGELLUM DEPENDENT CELL MOTILITY	GO:0001539	0,0094	-1,98
	CEREBROSPINAL FLUID CIRCULATION	GO:0090660	0,0089	-1,96
	REGULATION OF EXTRACELL. MATRIX ORGANIZATION	GO:1903053	0,0089	-1,95
	GLIAL CELL PROLIFERATION	GO:0014009	0,0085	-1,95
	EXTRACELLULAR MATRIX BINDING	GO:0050840	0,0194	-1,93
	INTEGRIN MEDIATED SIGNALING PATHWAY	GO:0007229	0,0134	-1,92
	CELL–CELL ADHESION MEDIATED BY INTEGRIN	GO:0033627	0,0169	-1,89
	MORPHOGENESIS OF A POLARIZED EPITHELIUM	GO:0001738	0,0165	-1,89

Repressed	CELL SUBSTRATE ADHESION	GO:0031589	0,0225	-1,86
	ASTROCYTE PROJECTION	GO:0097449	0,0367	-1,74
	ASTROCYTE END FOOT	GO:0097450	0,0414	-1,73

The table lists significantly enriched Gene Ontology (GO) biological processes identified in the OH vs WT comparison. Positively enriched (activated) and negatively enriched (repressed) pathways are shown with their corresponding GO identifiers, false discovery rate (FDR q-value), and normalized enrichment score (NES). Only gene sets with FDR q < 0.05 are included.

Table S3. *Gene Set Enrichment Analysis (GSEA) of periaqueductal tissue from AQP4⁻/⁻ mice without hydrocephalus (AQP4⁻/⁻-NH) compared to wild-type (WT) mice.*

NH vs WT				
	Description		FDR q-val	NES
Activated	MYELIN SHEATH	GO:0043209	< 1.0E-5	2,26
	PROTON TRANSPORTING V TYPE ATPASE COMPLEX	GO:0042776	< 1.0E-5	2,22
	SYNAPTIC VESICLE MATURATION	GO:0016188	0,0035	2,19
	MITOCHON. RESPIRATORY CHAIN COMPLEX ASSEMBLY	GO:0033108	0,0028	2,18
	ATP BIOSYNTHETIC PROCESS	GO:0006754	0,0202	2,02
	ATPASE ACTIVATOR ACTIVITY	GO:0001671	0,0234	2,02
	ATPASE COUPLED ION TRANSMEMBRANE TRANSPORTER ACTIVITY	GO:0042626	0,0258	2,00
	REACTIVE OXYGEN SPECIES BIOSYNTHETIC PROCESS	GO:1903409	0,0355	1,96
	MITOCHONDRIAL ELECTRON TRANSPORT NADH TO UBIQUINONE	GO:0006120	0,0344	1,96
	MICROGLIA DIFFERENTIATION	GO:0014004	0,0411	1,94
	NADH DEHYDROGENASE COMPLEX	GO:0030964	0,0155	1,92
	NEURON PROJECTION CYTOPLASM	GO:0120111	0,0232	1,89
	ATPASE COMPLEX	GO:1904949	0,0228	1,89
	AXON CYTOPLASM	GO:1904115	0,0298	1,84
	ATPASE DEPENDENT TRANSMEMBRANE TRANSPORT COMPLEX	GO:0098533	0,0311	1,83
	DISTAL AXON	GO:0150034	0,0418	1,79
Repressed	CILIARY PLASM	GO:0097014	0,0036	-2,10
	TASTE RECEPTOR ACTIVITY	GO:0008527	0,0084	-2,05
	CEREBROSPINAL FLUID CIRCULATION	GO:0090660	0,1254	-2,00
	MICROVILLUS MEMBRANE	GO:0031528	0,0124	-1,96
	CILIUM OR FLAGELLUM DEPENDENT CELL MOTILITY	GO:0001539	0,1219	-1,86
	NATURAL KILLER CELL CYTOKINE PRODUCTION	GO:0002370	0,1723	-1,81
	CELL ADHESION MOLECULE PRODUCTION	GO:0060352	0,2239	-1,77

The table lists significantly enriched Gene Ontology (GO) biological processes identified in the NH vs WT comparison. Activated and repressed pathways are shown with their respective GO identifiers, false discovery rate (FDR q-value), and normalized enrichment score (NES). Only gene sets with FDR q < 0.05 are included.
